# Preparation and qualification of internal rabies reference standards for use in the rabies rapid fluorescent focus inhibition test

**DOI:** 10.1038/s41598-020-66754-8

**Published:** 2020-06-18

**Authors:** Tatyana M. Timiryasova, Shekema A. Hodge, Lingyi Zheng, Amy Singer, Deanne Vincent, Mohammad Rahman, Celine Petit, Monique Brown

**Affiliations:** Sanofi Pasteur, 1 Discovery Drive, Swiftwater, PA 18370 USA

**Keywords:** Immunology, Vaccines

## Abstract

The World Health Organization (WHO) international standard rabies immune globulins (SRIGs) allow the standardisation of the cell-based rapid fluorescent-focus inhibition test (RFFIT) for rabies virus neutralising antibody measurement. SRIG stocks have been depleted. We describe the preparation and qualification of two internal rabies reference standards (IRRSs), calibrated against WHO SRIGs. Candidate IRRSs IMORAB2, from human rabies immunoglobulin; and GCIRAB1, from pooled serum samples from healthy adults immunised with licensed rabies vaccine, were generated. IRRSs were qualified for use in RFFIT based on pre-determined acceptance criteria. Unitage (IU/mL) was assigned using WHO-1 and WHO-2 SRIGs as calibrators. Geometric mean concentrations (GMCs) (% geometric coefficient of variation), calibrated against WHO-1 and WHO-2 SRIGs, were: 1.8 IU/mL (18.7%) and 1.5 IU/mL (17.8%) for IMORAB2; and 2.9 IU/mL (17.5%) and 2.5 IU/mL (16.7%), respectively, for GCIRAB1. We demonstrated IRRS specificity in competition studies using homologous (inactivated Pitman Moore rabies virus) and heterologous (inactivated vesicular stomatitis virus) antigens and acceptable accuracy/linearity of WHO SRIGs using IRRSs as calibrators. Concordance between IRRS and the WHO-1 SRIG was demonstrated using (non-)clinical human serum samples. The candidate reference standards are suitable for use as IRRS in the in-house rabies RFFIT. Funding:Sanofi Pasteur.

## Introduction

Rabies is a devastating disease and remains a major public health issue in regions such as Africa, Asia, Latin America and the Caribbean^1^. Left untreated, infection with the rabies virus (RABV), usually following a bite from an infected animal (often dogs), almost invariably results in fatal encephalitis with onset of disease symptoms. Globally, there are an estimated 59,000 human deaths annually, with 95% of cases occurring in Africa and Asia, predominantly among children living in poor rural communities^1^. However, the true burden of rabies is likely to be grossly underestimated due to misdiagnosis or a limited or poor surveillance infrastructure in most rabies-endemic regions^2^.

Protection against rabies can be achieved by induction of rabies virus neutralising antibodies (RVNA) through pre- or post-exposure vaccination with rabies vaccines^3^. Post-exposure vaccination by human or equine anti-rabies immunoglobulin injections around the wound site to inhibit viral spread, and with appropriate initial wound management, are collectively referred to as post-exposure prophylaxis (PEP). Measurement of RVNA levels is the standard method of confirming immunity to rabies following rabies pre-exposure vaccination or PEP^4^.

RVNA concentration ≥0.5 IU/mL is widely accepted as indicative of seroconversion following vaccination, and as an adequate proxy for protection^1,4^. Reliable detection and quantification of RVNA is thus essential in determining immunity among those who have undergone pre- or post-exposure vaccination against RABV, and in indirectly evaluating the effectiveness of PEP schedules. The rapid fluorescent focus inhibition test (RFFIT), a cell-based *in vitro* functional assay, is routinely used by World Health Organization (WHO) reference laboratories for assessment of RVNA in serum. Due to the inherent variability in the RFFIT, the RVNA titres are compared with titres achieved with WHO reference standard rabies immune globulins (SRIGs) with defined potency. Results are normalised to international units (IU)/mL to allow comparisons between laboratories and different assay formats.

The WHO-1 SRIG R-3 (59 IU/mL) and WHO-2 SRIG RAI (30 IU/mL), established in 1984 and 1994, respectively, have been used at the Sanofi Pasteur (SP) Global Clinical Immunology (GCI; Swiftwater, USA) department for measuring RVNA levels in clinical trial samples. The WHO-1 SRIG, the first rabies immunoglobulin reference serum of human origin, was prepared from pooled sera from vaccinated humans^5^. A second pool of sera from vaccinated humans was tested in 1993 (WHO-2)^6^. However, as stocks of the WHO-1 and WHO-2 SRIGs are now depleted, replacement rabies reference standard preparations are needed for testing. In this study, two potential internal rabies reference standards (IRRSs), IMORAB2 and GCIRAB1, were created and were calibrated against the WHO-1 and WHO-2 SRIGs for use in the SP in-house RFFIT. These new reference standard preparations ensure continued direct comparability of RVNA level measurements over time within the laboratory.

## Results

### Assignment of unit values

**Figure 1:**
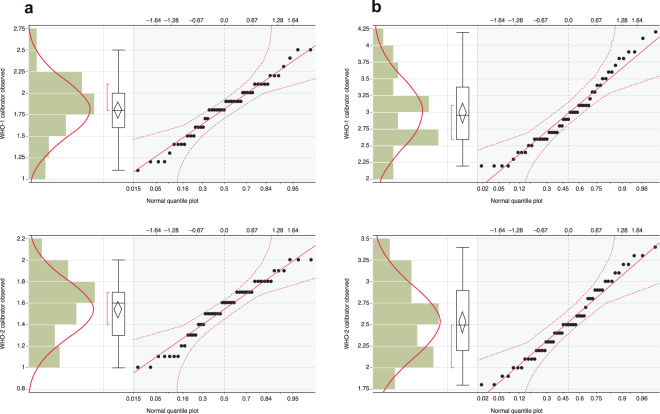
Histograms and normality assessment for assignment of unit values for candidate IRRSs (**a**) IMORAB2 and (**b**) GCIRAB1. These figures show observed results (y-axis; IU/mL) calibrated against the WHO-1 or WHO-2 SRIGs, visualised as histograms (left-hand panel), box and whiskers plot (median, interquartile ranges and range) and a normal quantile plot. SRIG, international standard rabies immune globulin; WHO, World Health Organization.

The observed geometric mean concentrations (GMCs) for IMORAB2 lot #1 were 1.8 IU/mL using WHO-1 SRIG as the calibrator and 1.5 IU/mL using WHO-2 SRIG as the calibrator (Table 1**;** Fig. 1a). For GCIRAB1, unit values were first assigned to the undiluted pooled preparation (Supplementary Table S1). Based on the assignment value of the undiluted material, GCIRAB1 was diluted to approximately 2.0 IU/mL. The observed GMCs for diluted GCIRAB1 lot #1 were 2.9 IU/mL using WHO-1 SRIG as the calibrator and 2.5 IU/mL using WHO-2 SRIG as the calibrator (Table 1**;** Fig. 1b). The unitage in IU/mL assigned using the WHO-1 SRIG was used for the qualification of the candidate IRRSs.

**Table 1:** Assignment of unit values for IMORAB2 and GCIRAB1.

IRRS Candidate	Calibrator	GMC (IU/mL)	Standard Deviation (SD)	Precision (%GCV)	Min (IU/mL)	Max (IU/mL)	Shapiro-Wilk Test (p-value)
IMORAB2 (N = 51)	WHO-1 SRIG	1.8	0.34	18.7%	1.1	2.5	0.3643
	WHO-2 SRIG	1.5	0.27	17.8%	1.0	2.0	0.0047
GCIRAB1 (N = 52)	WHO-1 SRIG	2.9	0.52	17.5%	2.2	4.2	0.0636
	WHO-2 SRIG	2.5	0.42	16.7%	1.8	3.4	0.1956

GCV, geometric coefficient of variance; GMC, geometric mean concentration; IU, international unit; N, number of valid runs used for the analysis; SD, standard deviation; SRIG, standard rabies immune globulin.

### Precision

Precision (measured as the % geometric coefficient of variation [GCV]) was in the acceptable range of ≤30% for both candidate IRRSs (Table 1).

### ED50 titres

**Figure 2:**
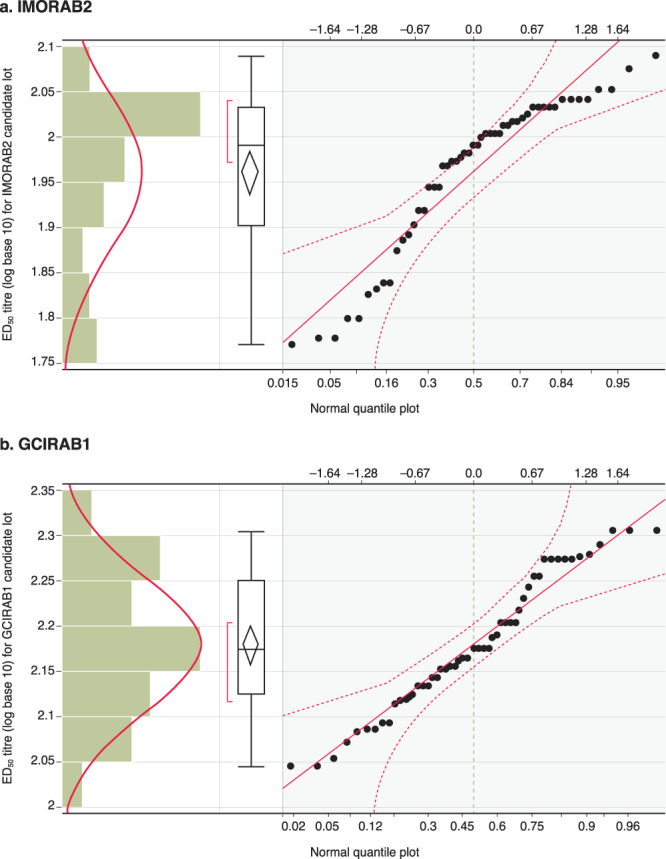
Histograms and normality assessment of ED_50_ titres for candidate IRRSs (**a**) IMORAB2 and (**b**) GCIRAB1. These figures show the 50% neutralisation point (y-axis; ED_50_) titres for IRRS candidates, visualised as histograms (left-hand panel), box and whiskers plot (median, interquartile ranges and range) and a normal quantile plot. ED_50_, 50% neutralisation point.

Geometric mean titres (GMTs) (1/dil) of 92 (lower limit, 62; upper limit, 136), with 21.9% precision for IMORAB2; and 151 (108; 212), with 18.5% precision for GCIRAB1, were established. The percentage of results within the acceptable range (± 2 standard deviation [SD]) was 94.1% for IMORAB2 and 100% for GCIRAB1 (Fig. 2**;** Supplementary Table S2).

### Specificity

**Figure 3:**
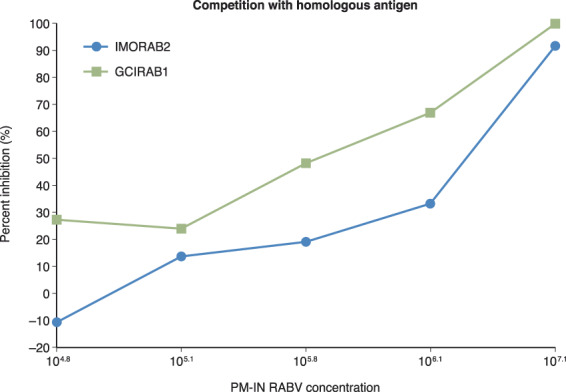
Specificity of IMORAB2 and GCIRAB1: dose-dependent inhibition with homologous competitor. Graphs show dose-dependent reductions in ED_50_ titres when tested against varying amounts of inactivated homologous antigen (PM-IN).A dose-dependent inhibition in the ED_50_ titers was observed when various amount of homologous competitor (PM-IN) was used. ED_50_, 50% neutralisation point, PM-IN RABV, inactivated Pitman Moore rabies virus.

Both candidate IRRSs showed a reduction in ED_50_ titres of ≥80% in the presence of the highest concentration of the homologous competitor inactivated Pitman-Moore (PM-IN) RABV. Reductions in ED_50_ titres were dose-dependent when tested against varying amounts of homologous competitor, showing that candidate IRRS are specific for RVNA and suitable as a calibrator for measuring RVNA in test human serum samples (Fig. 3). The presence of the heterologous antigen, inactivated vesicular stomatitis virus (VSV-IN), had limited impact (<30% change) on the ED_50_ titre, with 0.4% inhibition for IMORAB2 and 24.1% inhibition for GCIRAB1, in the presence of 10^3.0^ VSV-IN. Thus, both IMORAB2 and GCIRAB1 met the pre-defined acceptance criteria for specificity based on competition with homologous and heterologous antigens.

### Accuracy/linearity

**Figure 4:**
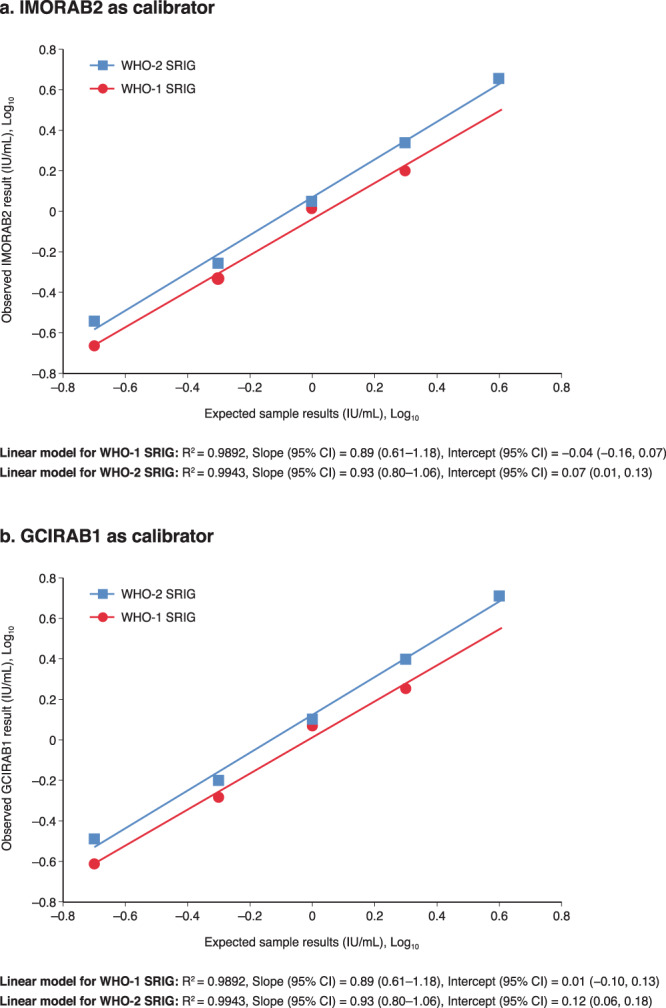
Regression analyses of observed versus expected geometric means concentrations for WHO-1 and WHO-2 SRIGs using candidate IRRSs as calibrator. These regression analyses demonstrate dilutional linearity of WHO-1 and WHO-2 SRIGs using (**a**) IMORAB2 and (**b**) GCIRAB1 as calibrators. IU, international unit; SRIG, standard rabies immune globulin; WHO, World Health Organization.

The acceptance criteria for the accuracy of WHO-1 and WHO-2 SRIGs using the candidate IRRSs as calibrators were met for both WHO-1 and WHO-2 SRIGs using results that were greater than or equal to LLOQ of 0.2 IU/mL: the percent recovery of the observed versus expected GMCs was within the range of 70–130% for 100% (4/4) of WHO-1 and 100% (5/5) of WHO-2 SRIG concentrations, using IMORAB2 as calibrator; and for 100% (4/4) of WHO-1 and 80% (4/5) of WHO-2 SRIG concentrations using GCIRAB1 as calibrator. These results are shown in Supplementary Table S3. Dilutional linearity of WHO-1 and WHO-2 SRIGs also met acceptance criteria using both IMORAB2 and GCIRAB1 as calibrators: regression slopes of observed versus expected GMCs were in the acceptable range of 0.80–1.25 and values for R^2^ were ≥0.95 (Fig. 4). The WHO-1 and WHO-2 SRIG samples with reported GMCs below the lower limit of quantitation (LLOQ) of 0.2 UI/mL and above the calculated endpoint were not included in the statistical analysis.

### LLOQ

Based on 12 RVNA-positive serum samples, %GCV of the intra-assay and intermediate precision was within the acceptable limit of <30% for both candidate IRRSs (IMORAB2: 13.8% [95% confidence interval (CI), 12.0; 16.2] for both intra-assay and intermediate precision; GCIRAB1: 12.3% [10.7; 14.5] for intra-assay and 14.1% [10.3; 22.2] for intermediate precision). The full set of results is shown in Supplementary Table S4. All four RVNA-negative serum samples tested negative in all three assay runs for both candidate IRRSs. Acceptance criteria for the LLOQ were met (Table 2); 0.2 IU/mL was confirmed as the LLOQ for the RFFIT assay for both candidate IRRSs.

**Table 2:** Acceptance criteria for each qualification parameter.

Parameter tested	Acceptance criteria
Assignment of unit value (IU/mL)	The precision should not exceed a GCV of 30%.
Establishment of ED_50_ titre range	The assigned range is the 10^(mean ± 2 SD)^ where mean and standard deviation (SD) are calculated using log_10_-transformed ED_50_ titres.
Specificity	*Competition with homologous antigen* The highest concentration of the homologous competitor must decrease the rabies virus neutralising antibody (RVNA) titre values by ≥80%. *Competition with heterologous antigen* Heterologous (unrelated) competitor must not cause the RVNA titre to reduce by>30% as compared to the RVNA titre obtained without any competition (baseline control).
Accuracy/linearity	80% of the spiked WHO-1 and WHO-2 SRIGs with results ≥ LLOQ, must have percent recovery of 70–130% for both WHO-1 and WHO-2 SRIGs. Linear regression slope (observed GMC vs. expected) must be 0.80–1.25 and the coefficient of determination (R^2^) must be ≥ 0.95 for both WHO-1 and WHO-2 SRIGs.
LLOQ	The calculated %GCV for the positive samples near LLOQ must be ≤30%. All negative serum must remain negative.
Concordance	The 90% confidence interval (CI) of the concordance slope must be within the range of 0.80–1.25. The 90% CI of the percent difference must be within the range (−30%, 30%).

CI, confidence interval; ED_50_, 50% neutralisation point; GCV, geometric coefficient of variance; GMC, geometric mean concentration; IU, international unit; LLOQ, lower limit of quantitation; RVNA, rabies virus neutralising antibody; SRIG, standard rabies immune globulin.

### Concordance

**Figure 5:**
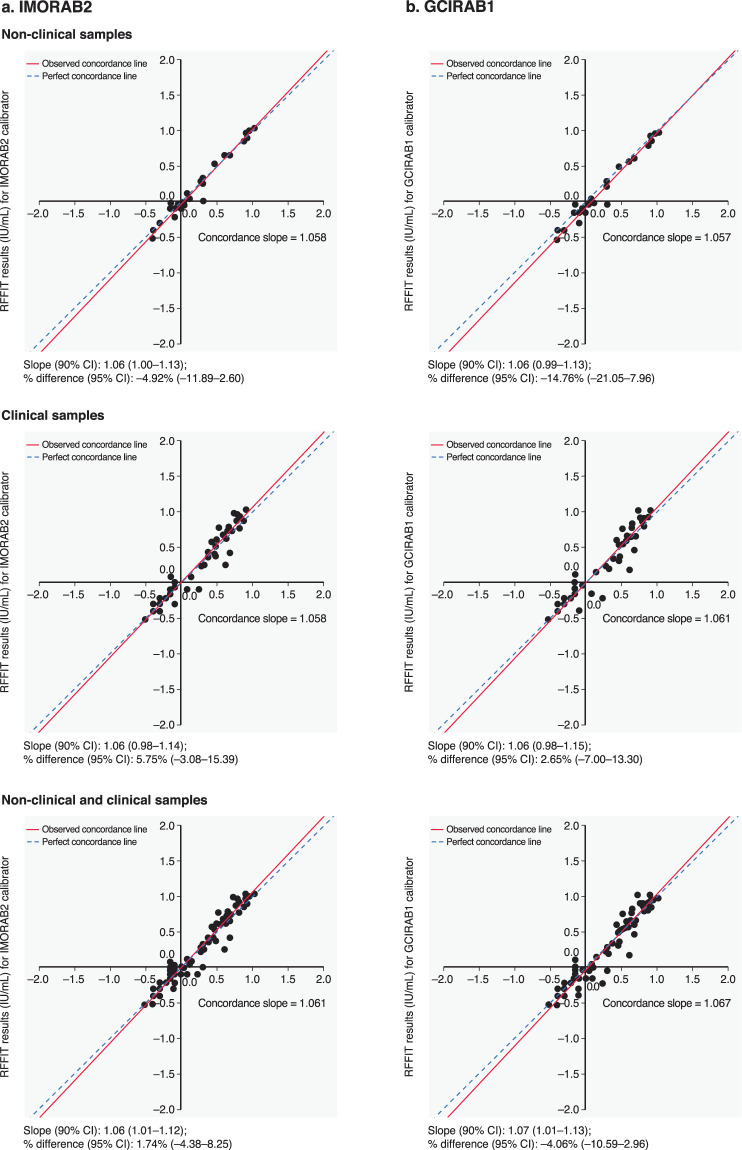
Evaluation of concordance of (**a**) IMORAB2 and (**b**) GCIRAB1 IRRSs with WHO-1 calibrator using clinical and non-clinical samples. These figures show a statistical relationship between candidate IRRSs and the WHO-1 SRIG using non-clinical, clinical and combined (non-clinical and clinical) samples. Concordance was demonstrated based on the 90% confidence intervals (CI) of the concordance slope and of the percent difference in GMC values meeting pre-determined acceptance criteria. CI, confidence interval; IU, international unit; RFFIT, rapid fluorescent focus inhibition test.

The 90% CI of the concordance slope was within the acceptable interval of 0.80–1.25 for clinical and non-clinical samples, and clinical and non-clinical samples combined, for both candidate IRRSs (Fig. 5). The 90% CI of the percent differences in the RFFIT GMC values between those generated using the WHO-1 SRIG and those generated with IMORAB2 or GCIRAB1 as calibrator were within the acceptable range, of −30% to 30% (Fig. 5). Evaluation of serostatus at a threshold titre of 0.5 IU/mL showed agreement between the WHO-1 SRIG and IMORAB2 for all non-clinical samples (30/30), 96.7% (58/60) of clinical samples and 97.8% (88/90) of non-clinical and clinical samples combined. For GCIRAB1, agreement with WHO-1 SRIG was observed for 96.7% (29/30), 95.0% (57/60), and 95.6% (86/90) of non-clinical, clinical, and non-clinical and clinical samples combined, respectively. GMC ratios (95% CI) for IMORAB2 vs WHO-1 SRIG were 0.95 (0.88; 1.03) for non-clinical samples (n = 28), 1.06 (0.97; 1.15) for clinical samples (n = 49) and 1.02 (0.96; 1.08) for non-clinical and clinical samples combined (n = 77); for GCIRAB1 vs WHO-1 SRIG, GMC ratios were 0.85 (0.79; 0.92) for non-clinical samples, 1.03 (0.93; 1.13) for clinical samples and 0.96 (0.89; 1.03) for non-clinical and clinical combined (Supplementary Table S5).

## Discussion

The use of a reference standard in laboratory assays is essential for the calibration and harmonization of assay data, allowing for meaningful interpretation of results and comparisons between laboratories and between studies over time^7–10^. The RFFIT assay is complex due to the fact that sensitivity and specificity are affected by multiple factors, lending to potential variability between results obtained from different laboratories. The harmonization of the RFFIT is particularly important as the originally described method^11^ has been modified such that different assay formats are currently used^12–14^.

In this study, candidate IRRSs, IMORAB2 and GCIRAB1, were prepared and calibrated using the WHO-1 and WHO-2 SRIGs, for use in the SP in-house rabies RFFIT method. Pre-defined acceptance criteria were met for the qualification of both candidate IRRSs, which included: assessment of the precision of the assigned unitage and established ED_50_ titre ranges; specificity of the IRRSs in competition with homologous and heterologous antigens; the accuracy and dilutional linearity of the WHO SRIGs when using the candidate IRRSs as calibrators; and evaluation of the concordance of results obtained with the IRRSs with those obtained using the international WHO-1 SRIG. Indeed, results using IMORAB2 and GCIRAB1, with either clinical or non-clinical samples, showed good concordance with historical data generated during a previous rabies vaccine clinical trial or through prior sample testing using WHO-1 as a calibrator.

The reference standard should reflect how a test sample would behave in the assay, across a range of assay formats^10^. Pooled serum samples from several individuals who have been exposed to the pathogen, via infection or vaccination, provide a widened spectrum of antibody reactivates and more closely represent a test sample matrix. As such, they are more likely to work in a range of assay formats and are thus considered to be an ideal basis for an antibody assay standard^10^. While our results showed that the GCIRAB1, generated from pooled sera, is suitable for use as an internal reference standard for RVNA testing with the RFFIT, we also showed that the candidate IRRS prepared from commercially available human rabies immunoglobulin would be suitable for use as well. The use of purified rabies immunoglobulin rather than serum samples would reduce the levels of biosafety containment required for handling samples in the laboratory. Although it is feasible to obtain rabies immunoglobulin, it’s matrix would in theory be more limited than that of pooled sera^10^.

The assigned values for the potency of the WHO-1 and WHO-2 SRIGs (59 and 30 IU/mL, respectively) are important in providing known values against which we were able to calibrate the candidate IRRSs for use in standardized in-house testing. As they are not exactly equivalent and have both been used extensively in different studies, both WHO SRIGs were used for calibration of the candidate IRRSs to allow for comparability.

The importance of the WHO recommendation to use a single recognized reference standard for assay validation^13^ has been shown in a previous study comparing RFFIT performance with different standard sera. Titres obtained using standards prepared by the National Institute for Food and Drug Control of China were significantly different to titres obtained using WHO international standards^15^. It should also be noted that the potency assigned to a reference standard by one method, e.g. the RFFIT, may not be the same in a different method such as an ELISA method^16^. The unit values assigned for the candidate reference standards described will thus be applicable specifically to the RFFIT method in which it was qualified.

The shelf-life and stability of the reference standard are important aspects to be considered and ideally optimized such that assay performance can be monitored with the same standard over a number of years^17^. However, the degradation of prepared references can occur over time despite being stored under controlled conditions. The stability of the internal references evaluated in this study will therefore need to be monitored by RFFIT testing, with results compared to pre-established baseline results, using in-run controls and performance monitoring of sample panels to monitor any drift over time. Regular monitoring of assay performance using the reference standard and pre-defined criteria is also essential to avoid drift in assay performance, and to control for potential modifications over time^10,13^.

## Conclusion

In the current study, we demonstrated the evaluation and qualification of two IRRSs, prepared using two different approaches. Both the candidate IRRSs IMORAB2, prepared from human rabies immunoglobulin, and GCIRAB1, prepared from pooled RVNA positive human serum samples, met pre-determined acceptance criteria. These reagents are both suitable for use as internal reference standards in the SP in-house rabies RFFIT method; their use will ensure continued direct comparability of RVNA level measurements over time, and between studies, within the laboratory.

## Methods

### Preparation of IRRSs

Two different approaches were used for the preparation of the IRRSs. IMORAB2 was prepared from human rabies immunoglobulin produced by SP (Imogam, 187 IU/mL) that was diluted with serum-free cell culture medium to a target GMC of approximately 2.0 IU/mL (a total volume of approximately 140 mL was prepared). The diluted candidate IRRS was assigned a lot number (e.g., lot #1) and aliquoted at 240 μL/vial for storage at −40 °C to −80 °C; the stability and performance of the diluted material was monitored in each RFFIT assay run.

GCIRAB1 was prepared from pooled human serum samples positive for RVNA. Samples from nine donors vaccinated with the licensed human diploid cell rabies vaccine (Imovax Rabies) several years before sample donation were screened using the in-house RFFIT; four donors were selected based on the level of RVNA in the sample. Selected samples were pooled and aliquoted (undiluted) at 5 mL/tube and stored at −40 °C to −80 °C (total volume, 250 mL). 24 mL of GCIRAB1 (undiluted) was diluted with serum-free cell culture medium to a target GMC of approximately 2.0 IU/mL. The diluted candidate IRRS was assigned a lot number (e.g., lot #1) and aliquoted at 240 μL/vial for storage at −40 °C to −80 °C; the stability and performance of the diluted material was monitored in each RFFIT assay run.

All experiments and experimental protocols conducted to evaluate and qualify the IRRS were approved by SP, Swiftwater, PA.

### RFFIT method

The RFFIT, initially described by Smith *et al*. (1973)^11^, was implemented under optimised and validated conditions^18^. The RVNA titre (50% neutralisation, ED_50_) of a test sample was mathematically interpolated using the Reed and Muench method^19^ and was calibrated and converted into IU/mL against the WHO SRIG or the candidate IRRS.

### Cells and rabies virus

Baby hamster kidney (BHK)-21 cell banks (American Type Culture Collection, catalog #CL-10) were qualified before use in in-house RFFIT and cultured as described previously^18^. Challenge virus standard 11 (CVS-11), lot #MLE 04–2015, was produced by SP at Marcy L’Etoile using virus stock received from Centers for Disease Control and Prevention, Atlanta. The RABV CVS-11 strain was diluted to target a challenge virus dose of 50 (tissue culture infectious dose [TCID]_50_)/100 μL and back-titrated to determine the actual TCID_50_ for each assay run and was qualified for use in in-house RFFIT as described previously^18,19^.

### WHO SRIGs

The WHO-1 SRIG (lot R-3; 59 IU/mL) was obtained from the Food and Drug Administration/Center for Biologics Evaluation and Research; WHO-2 SRIG (lot RAI; 30 IU/mL) was obtained from the National Institute for Biological Standards and Controls. Both were diluted to 2.0 IU/mL according to manufacturer’s instructions, and heat inactivated at 56 °C for 30 min prior to use.

### Test serum samples

Human serum samples were obtained from healthy adult SP employees: RVNA-positive samples were obtained from those immunised with human diploid cell vaccine (Imovax Rabies); RVNA-negative samples were obtained from unvaccinated individuals. Samples were prepared, coded, and randomised (blinded) by a biostatistician. Procedures complied with Health Insurance Portability and Accountability regulations and Sanofi Policies and Procedures. Informed consent was obtained from these volunteers to collect samples for research use. Twelve 150 μL single-use aliquots were created for each sample, and heat inactivated at 56 °C for 30 min before use.

An additional subset of clinical serum samples from individuals who had participated in a previous SP purified Vero rabies vaccine clinical study (NCT03145766), was used for concordance studies. The study was carried out in accordance with the Declaration of Helsinki and the International Conference on Harmonisation guidelines for Good Clinical Practice. Written informed consent to use clinical samples for future research purposes was obtained during the clinical trial following the study protocol approval by the local Institutional Review Board (IRB). Only those samples for which participants had provided informed consent for future research use were included in the current study. Sixty samples with RVNA titres ranging from <0.2 IU/mL to 8.0 IU/mL were prepared, coded and randomised by biostatisticians for use in this study. Samples were heat inactivated at 56 °C for 30 min prior to use.

### Qualification of IRRSs

#### Assignment of unit value (IU/mL)

IRRS candidates were tested in multiple assay runs by multiple qualified analysts to generate >50 results, using the WHO-1 or WHO-2 SRIGs as calibrators. The GMC obtained using the WHO-1 SRIG and WHO-2 SRIG as the calibrator was reported as the unit value. The unit (IU/mL) assigned using the WHO-1 SRIG was used for IRRS candidate qualification. Acceptance criteria for assignment of unit values are shown in Table 2.

#### Assessment of precision

Precision was assessed by calculation of the GCV using variance component analysis; %GCV = $100 \% \times ({{\rm{e}}}^{{\rm{\sigma }}}-1)$, where the GCV estimate $({{\rm{e}}}^{{\rm{\sigma }}}-1)$ is thus assessed using a sample standard deviation ${\rm{\sigma }}$^ to estimate σ, where σ^ is obtained from variance component analysis of the log transformed results of the precision experiment.

The normal distribution of the data was verified using the Shapiro-Wilk test. The data were considered normal if the test failed to reject non-normality (p-value <0.05); in this case, the arithmetic mean and percent of coefficient of variation (%CV) were estimated. If the test rejected normality, then the data were log_10_-transformed prior to estimation of the geometric mean and the percent of GCV.

#### Establishing the ED_50_ titre range

For each IRRS candidate diluted to approximately 2.0 IU/mL, valid ED_50_ titres (51 results for IMORAB2; 52 results for GCIRAB1) were generated in multiple assay runs. The GMT of the observed ED_50_ titres (1/dil) was calculated and precision (%GCV) assessed. The Shapiro-Wilk test was used to verify the normal distribution of the data. Acceptance criteria for the range of ED_50_ titres are shown in Table 2.

#### Specificity

Specificity was assessed in competition studies using the homologous antigen PM-IN RABV (SP, Marcy L’Etoile lot #FA500452), at a concentration of 10^8.8^ 50% cell culture infectious dose (CCID_50_)/mL before inactivation, and heterologous antigen VSV-IN (Kansas State Veterinary Diagnostic Laboratory) at a concentration of 10^7^ TCID_50_/mL before inactivation. Both antigens have been used previously for RFFIT assay validation in accordance with ICH guidelines^16,18^. The candidate IRRS was competed with 10^7.1^, 10^6.1^, 10^5.8^, 10^5.1^, and 10^4.8^ CCID_50_/0.1 mL of PM-IN competitor and 10^3.0^ TCID_50_/0.1 mL of the VSV-IN competitor or competed with assay medium (baseline control). For the purpose of this study, IMORAB2 was diluted to approximately 9.0 IU/mL and GCIRAB1 to approximately 7.0 IU/mL. The specificity of the IRRS candidate was evaluated as the % reduction (or difference) of the observed ED_50_ titre in the presence of competitors as compared to the expected ED_50_ titres in the absence of competitors (baseline control). The competed IRRS samples were tested in one assay run. Acceptance criteria are defined in Table 2.

#### Accuracy/linearity

The WHO-1 and WHO-2 SRIGs were spiked into a RVNA-negative human serum sample matrix at different concentrations: WHO-1 at five different concentrations (2.0, 1.0, 0.5, 0.2, and 0.1 IU/mL) and WHO-2 at seven concentrations (8.0, 4.0, 2.0, 1.0, 0.5, 0.2, and 0.1 IU/mL). The dilution scheme for the WHO-1 SRIG included only five different concentrations because the original stock was depleted and the highest concentration available for testing was 2.0 IU/mL. The SRIGs at each concentration were tested five times in one assay run and the GMC was calculated. Only samples with a concentration of ≥0.2 IU/mL were used in the analysis.

Accuracy was analysed for each concentration as the percent (%) recovery of the observed GMC relative to the expected GMC; dilutional linearity for accuracy was also assessed by a linear regression analysis of the observed and expected GMC for all concentrations of the SRIGs. Acceptance criteria for evaluation of accuracy and linearity are shown in Table 2.

#### Lower limit of quantitation

The LLOQ of the RFFIT was evaluated using 12 serum samples with a low concentration of RVNA ( ≤ 1.0 IU/mL) and 4 RVNA-negative serum samples. Each sample was tested in triplicate, in three independent assay runs, and GMC was calculated. The LLOQ was assessed based on the %GCV. Samples with a GMC < 0.2 IU/mL were reported to be <LLOQ and were not included in the calculation of %GCV. Acceptance criteria for the evaluation of the LLOQ are shown in Table 2.

#### Concordance

Concordance between each candidate IRRS and the WHO-1 SRIG was evaluated using a panel of human serum samples consisting of 30 non-clinical (from SP-employee donors, including 28 RVNA-positive and 2 RVNA-negative samples) and 60 clinical samples (including 10 RVNA-negative samples [<0.2 IU/mL], and 50 RVNA-positive samples).

Non-clinical samples were tested in three independent assay runs, and clinical samples in two independent runs; GMCs were calculated for each sample and compared to historical target results, generated using the WHO-1 SRIG as the calibrator. Concordance slope and percentage differences were calculated.

Acceptance criteria for the evaluation of concordance are shown in Table 2.

### Statistical analysis

Analyses were descriptive. No hypothesis testing was undertaken.

## Supplementary information

Supplementary Information.

## Data Availability

The datasets generated and/or analysed during the current study are available from the corresponding author on reasonable request.
