# Supplementary material for: Preparation and qualification of internal rabies reference standards for use in the rabies rapid fluorescent focus inhibition test
Source: Sci Rep. 2020 Jun 18;10:9893. doi: 10.1038/s41598-020-66754-8 (PMC7303118; doi:10.1038/s41598-020-66754-8)
Supplement: Supplementary file 1 — Supplementary Information. [file 41598_2020_66754_MOESM1_ESM.docx]

Preparation and qualification of internal rabies reference standards for use in the rabies rapid fluorescent focus inhibition test

Tatyana M. Timiryasova,* Shekema A. Hodge, Lingyi Zheng, Amy Singer, Deanne Vincent, Mohammad Rahman, Celine Petit, and Monique Brown

Sanofi Pasteur, 1 Discovery Drive, Swiftwater, PA, 18370, USA

**Supplementary material**

**Supplementary Tables**

**Supplementary Table S1 Assignment of Unit for GCIRAB1 (undiluted)**

| **Calibrator** | **N** | **GMC (IU/mL)** | **Standard Deviation** | **Precision (%GCV)** | **Min (IU/mL)** | **Max (IU/mL)** | ***p-*value of Shapiro- Wilk Test** |
| --- | --- | --- | --- | --- | --- | --- | --- |
| **WHO-1 SRIG** | 50 | 11.7 | 1.4 | 12.1% | 8.8 | 16.2 | 0.3309 |
| **WHO-2 SRIG** | 60 | 10.4 | 1.3 | 12.8% | 6.8 | 15.0 | 0.0011 |

GCV, geometric coefficient of variance; GMC, geometric mean concentration; IU, international unit; N, number of results used for statistical analysis; SRIG, standard rabies immune globulin

**Supplementary Table S2. Establishment of ED_50_ titer range**

| **IRRS Candidate** | **GMT**  **(ED_50_, 1/Dil)** | **Precision**  **(%GCV)** | **Lower Limit** | **Upper Limit** | **% of results within the acceptable limits (n/N)** |
| --- | --- | --- | --- | --- | --- |
| **IMORAB2 (N=51)** | 92 | 21.9% | 62 | 136 | 94.1% (48/51) |
| **GCIRAB1 (N=52)** | 151 | 18.5% | 108 | 212 | 100% (52/52) |

ED_50_, 50% neutralization point; GCV, geometric coefficient of variance; GMT, geometric mean titer; N, number of valid results used for the analysis; n, number of results within the established range

**Supplementary Table S3. Accuracy of WHO-1 and WHO-2 SRIGs using IMORAB2 of GCIRAB1**

|  | **IMORAB2** | | **GCIRAB1** | |
| --- | --- | --- | --- | --- |
| **Expected concentration (IU/mL)** | **Observed GMC vs IRRS (IU/mL)** | **Percent recovery (%)** | **Observed GMC vs IRRS (IU/mL)** | **Percent recovery (%)** |
| **WHO-1 SRIG** | | | | |
| **2.0** | 1.576 | 78.8% | 1.782 | 89.1% |
| **1.0** | 1.035 | 103.4% | 1.170 | 117.0% |
| **0.5** | 0.460 | 92.0% | 0.520 | 104.0% |
| **0.2** | 0.215 | 107.7% | 0.244 | 121.8% |
| **0.1** | 0.132† | N/A | 0.149† | N/A* |
| **WHO-2 SRIG** | | | | |
| **8.0** | 13.755†† | N/E | 15.546†† | N/E |
| **4.0** | 4.559 | 114.0% | 5.153 | 128.8% |
| **2.0** | 2.185 | 109.3% | 2.470 | 123.5% |
| **1.0** | 1.108 | 110.8% | 1.253 | 125.3% |
| **0.5** | 0.555 | 111.0% | 0.627 | 125.5% |
| **0.2** | 0.247 | 123.3% | 0.278 | 139.2% |
| **0.1** | 0.119† | N/A | 0.134† | N/A |

†The reported values and GMC were below LLOQ (i.e., < 0.2) and were not used for statistical analysis.

††The reported values were “Retest High” and were not used for statistical analysis.

GCV, geometric coefficient of variance; GMC, geometric mean concentration; IU, international unit; LLOQ, lower limit of quantitation; N/A, not applicable; N/E, not estimated; SRIG, standard rabies immune globulin

**Supplementary Table S4. RFFIT LLOQ results using IMORAB2 and GCIRAB1**

| Test Samples | **Run #1** | | | **Run #2** | | **Run #3** | | | **GMC of 3 Runs (IU/mL)** | **%GCV**  **of 3 Runs** | |
| --- | --- | --- | --- | --- | --- | --- | --- | --- | --- | --- | --- |
|  | **GMC**  **IU/mL** | | **%GCV** | **GMC**  **IU/mL** | **%GCV** | **GMC**  **IU/mL** | **%GCV** | |  |  |  |
| **IMORAB2** | | | | | | | | | | | |
| 1 | 0.480 | 2.5% | | 0.665 | 0.5% | 0.484 | | 2.3% | 0.536 | 17.6% | |
| 2 | 0.498 | 4.0% | | 0.621 | 2.4% | 0.497 | | 4.6% | 0.535 | 12.2% | |
| 3 | 0.541 | 3.8% | | 0.684 | 6.0% | 0.567 | | 8.5% | 0.594 | 12.8% | |
| 4 | 0.372 | 5.5% | | 0.480 | 11.0% | 0.419 | | 14.6% | 0.422 | 15.3% | |
| 5 | 0.316 | 12.1% | | 0.401 | 9.0% | 0.255 | | 5.7% | 0.319 | 23.5% | |
| 6 | 0.623 | 11.9% | | 0.674 | 2.3% | 0.501 | | 1.3% | 0.595 | 15.6% | |
| 7 | 0.557 | 4.5% | | 0.599 | 7.6% | 0.645 | | 4.4% | 0.599 | 8.2% | |
| 8 | 0.621 | 7.3% | | 0.680 | 2.2% | 0.665 | | 16.8% | 0.655 | 10.0% | |
| 9 | 0.506 | 4.4% | | 0.571 | 10.8% | 0.480 | | 2.9% | 0.517 | 10.1% | |
| 10 | 0.291 | 2.1% | | 0.340 | 3.2% | 0.273 | | 3.2% | 0.300 | 10.6% | |
| 11 | 0.616 | 13.3% | | 0.703 | 4.6% | 0.565 | | 4.4% | 0.626 | 12.5% | |
| 12 | 0.340 | 16.5% | | 0.338 | 5.7% | 0.365 | | 13.4% | 0.345 | 11.1% | |
| 13 | <0.2 | N/E | | <0.2 | N/E | <0.2 | | N/E | <0.2 | N/E | |
| 14 | <0.2 | N/E | | <0.2 | N/E | <0.2 | | N/E | <0.2 | N/E | |
| 15 | <0.2 | N/E | | <0.2 | N/E | <0.2 | | N/E | <0.2 | N/E | |
| 16 | <0.2 | N/E | | <0.2 | N/E | <0.2 | | N/E | <0.2 | N/E | |
| **GCIRAB1** | | | | | | | | | | | |
| 1 | 0.531 | 2.6% | | 0.492 | 0.5% | 0.436 | | 2.3% | 0.485 | | 9.1% |
| 2 | 0.551 | 3.9% | | 0.459 | 2.4% | 0.447 | | 4.6% | 0.484 | | 11.0% |
| 3 | 0.583 | 3.8% | | 0.654 | 6.0% | 0.511 | | 8.5% | 0.580 | | 12.7% |
| 4 | 0.401 | 5.5% | | 0.459 | 11.1% | 0.378 | | 14.7% | 0.411 | | 13.3% |
| 5 | 0.341 | 12.0% | | 0.384 | 9.0% | 0.230 | | 5.6% | 0.311 | | 27.7% |
| 6 | 0.671 | 12.0% | | 0.644 | 2.3% | 0.453 | | 1.3% | 0.580 | | 21.7% |
| 7 | 0.601 | 4.6% | | 0.573 | 7.6% | 0.674 | | 4.5% | 0.614 | | 9.1% |
| 8 | 0.670 | 7.4% | | 0.650 | 2.3% | 0.695 | | 16.9% | 0.671 | | 9.6% |
| 9 | 0.545 | 4.3% | | 0.546 | 10.8% | 0.502 | | 2.8% | 0.530 | | 7.4% |
| 10 | 0.314 | 2.3% | | 0.325 | 3.1% | 0.285 | | 3.3% | 0.307 | | 6.6% |
| 11 | 0.664 | 13.3% | | 0.673 | 4.7% | 0.591 | | 4.2% | 0.642 | | 9.8% |
| 12 | 0.367 | 16.4% | | 0.323 | 5.6% | 0.381 | | 13.4% | 0.353 | | 13.3% |
| 13 | <0.2 | N/E | | <0.2 | N/E | <0.2 | | N/E | <0.2 | | N/E |
| 14 | <0.2 | N/E | | <0.2 | N/E | <0.2 | | N/E | <0.2 | | N/E |
| 15 | <0.2 | N/E | | <0.2 | N/E | <0.2 | | N/E | <0.2 | | N/E |
| 16 | <0.2 | N/E | | <0.2 | N/E | <0.2 | | N/E | <0.2 | | N/E |

GCV, geometric coefficient of variance; GMC, geometric mean concentration; IU, international unit; N/E, not estimated

**Supplementary Table S5: GMC ratio and 95%CI for IMORAB2 and GCIRAB1**

|  | **Parameterr** | **Estimate** | **95% LCL** | **95% UCL** |
| --- | --- | --- | --- | --- |
| **IMORAB2** |  |  |  |  |
| Non-clinical samples (n=28) | GMC WHO-1 SRIG | 1.66 | 1.14 | 2.43 |
|  | GMC IMORAB2 | 1.58 | 1.06 | 2.36 |
|  | GMC Ratio (IMORAB2/WHO-1) | 0.95 | 0.88 | 1.03 |
| Clinical samples (n=49) | GMC WHO-1 SRIG | 1.76 | 1.32 | 2.36 |
|  | GMC IMORAB2 | 1.86 | 1.37 | 2.53 |
|  | GMC Ratio (IMORAB2/WHO-1) | 1.06 | 0.97 | 1.15 |
| Non-clinical and clinical samples (n=77) | GMC WHO-1 SRIG | 1.73 | 1.38 | 2.16 |
|  | GMC IMORAB2 | 1.76 | 1.38 | 2.23 |
|  | GMC Ratio (IMORAB2/WHO-1) | 1.02 | 0.96 | 1.08 |
| **GCIRAB1** |  |  |  |  |
| Non-clinical samples (n=28) | GMC WHO-1 SRIG | 1.66 | 1.14 | 2.43 |
|  | GMC GCIRAB1 | 1.42 | 0.95 | 2.12 |
|  | GMC Ratio (GCIRAB1/WHO-1) | 0.85 | 0.79 | 0.92 |
| Clinical samples (n=49) | GMC WHO-1 SRIG | 1.76 | 1.32 | 2.36 |
|  | GMC GCIRAB1 | 1.81 | 1.33 | 2.46 |
|  | GMC Ratio (GCIRAB1/WHO-1) | 1.03 | 0.93 | 1.13 |
| Non-clinical and clinical samples (n=77) | GMC WHO-1 SRIG | 1.73 | 1.38 | 2.16 |
|  | GMC GCIRAB1 | 1.66 | 1.30 | 2.10 |
|  | GMC Ratio (GCIRAB1/WHO-1) | 0.96 | 0.89 | 1.03 |

GMC, geometric mean concentration; IU, international unit; LCL, lower confidence limit; n, number of samples used for statistical analysis; SRIG, standard rabies immune globulin; UCL, upper confidence limit
